# Supplementary material for: Compounded environmental health risks in mountain communities upstream of Bhumibol Dam, Thailand
Source: PLoS One. 2026 Jun 9;21(6):e0340557. doi: 10.1371/journal.pone.0340557 (PMC13249165; doi:10.1371/journal.pone.0340557)
Supplement: S1 Table — (DOCX) [file pone.0340557.s001.docx]

**Supporting information**

**S1 Table.** Descriptive analysis of water quality parameters of samples from a community water filters (Village V3, Hin Lat), evaluated across three sampling points over 6 months at approximately 3-month intervals.

| **Tested variables** | **Timepoint 1** | | **Timepoint 2** | | **Timepoint 3** | | **Standard values** | **Method detection limit** | **Unit** | **Testing methods** |
| --- | --- | --- | --- | --- | --- | --- | --- | --- | --- | --- |
|  | **22 Nov, 2024** | | **4 Feb, 2025** | | **16 May, 2025** | |  |  |  |  |
|  | **Pre-filter** | **Post- filter** | **Pre-filter** | **Post- filter** | **Pre-filter** | **Post- filter** |  |  |  |  |
| Apparent colour | 4 | 2 | 6 | 4 | 3 | 0 | <15 | - | Pt-Co | #APHA, AWWA, WEF, 24th Edition, 2023. Part 2120 B. |
| Turbidity | 1.28 | 0.39 | 0.58 | 0.6 | 0.416 | 0.149 | <5 | - | NUT | #APHA, AWWA, WEF, 24th Edition, 2023. Part 2130 B. |
| pH at 25°C | 8.1 | 8 | 8.1 | 8.4 | 8.18 | 7.91 | 6.5-8.5 | - | - | #APHA, AWWA, WEF, 24th Edition, 2023. Part 4500-H+ B. |
| Total dissolved solids | 296 | 349 | 218 | 235 | 342 | 221 | <500 | 3.5 | mg/L | In-house method TM-CH-03 based on #APHA, AWWA, WEF, 24th Edition, 2023. Part 2540 C. |
| Hardness as CaCO_3_ | 243.7 | 307.3 | 166 | 178.7 | 312 | 205 | <300 | 0.8 | mg/L | In-house method TM-CH-02 based on #APHA, AWWA, WEF, 24th Edition, 2023. Part 2340 C. |
| Sulfate | 6 | 5 | 7 | 7 | 7 | 8 | <250 | 0.09 | mg/L | In-house method TM-CH-04 based on #APHA, AWWA, WEF, 24th Edition, 2023. Part 4110 C. |
| Chloride | <2 | <2 | 2 | 2 | 0 | 0 | <250 | 0.04 | mg/L | In-house method TM-CH-04 based on #APHA, AWWA, WEF, 24th Edition, 2023. Part 4110 C. |
| Nitrate as NO­­^−^_3_ | <1.0 | <1.0 | ND | <1.0 | <0.40 | 0.66 | <50 | 0.07 | mg/L | In-house method TM-CH-04 based on #APHA, AWWA, WEF, 24th Edition, 2023. Part 4110 C. |
| Fluoride | 0.25 | 0.21 | 0.26 | 0.26 | 0.19 | 0.2 | <0.7 | 0.008 | mg/L | In-house method TM-CH-04 based on #APHA, AWWA, WEF, 24th Edition, 2023. Part 4110 C. |
| Nitrite as NO^−^_3_ | <0.02 | ND | ND | ND | <0.007 | <0.007 | <3 | - | mg/L | #APHA, AWWA, WEF, 24th Edition, 2023. Part 4500-NO-2 B. |
| Iron | <0.010 | <0.010 | <0.010 | 0.021 | 0.01 | ND | <0.3 | 0.0021 | mg/L | In-house method TM-CH-05 based on #APHA, AWWA, WEF, 24th Edition, 2023. Part 3120 C. |
| Manganese | ND | ND | ND | <0.010 | 0.04 | 0.02 | <0.3 | 0.0006 | mg/L | In-house method TM-CH-05 based on #APHA, AWWA, WEF, 24th Edition, 2023. Part 3120 C. |
| Copper | ND | ND | ND | ND | <0.05 | <0.05 | <1.0 | 0.003 | mg/L | In-house method TM-CH-05 based on #APHA, AWWA, WEF, 24th Edition, 2023. Part 3120 C. |
| Zinc | 0.011 | 0.01 | <0.010 | 0.016 | 0.0685 | <0.01 | <3.0 | 0.003 | mg/L | In-house method TM-CH-05 based on #APHA, AWWA, WEF, 24th Edition, 2023. Part 3120 C. |
| Lead | ND | ND | ND | ND | ND | ND | <0.01 | - | mg/L | #APHA, AWWA, WEF, 24th Edition, 2023. Part 3125 B. |
| Total Chromium | ND | ND | ND | ND | <0.0005 | <0.0005 | <0.05 | - | mg/L | #APHA, AWWA, WEF, 24th Edition, 2023. Part 3125 B. |
| Cadmium | ND | ND | ND | ND | ND | ND | <0.003 | - | mg/L | #APHA, AWWA, WEF, 24th Edition, 2023. Part 3125 B. |
| Arsenic | ND | ND | ND | ND | <0.0005 | 0.0026 | <0.01 | - | mg/L | #APHA, AWWA, WEF, 24th Edition, 2023. Part 3125 B. |
| Mercury | ND | ND | ND | ND | ND | ND | <0.001 | - | mg/L | #APHA, AWWA, WEF, 24th Edition, 2023. Part 3125 B. |
| Coliform bacteria | >23 | >23 | >23 | >23 | 23 | >23 | <1.1 | - | MPN/ 100 mL | #APHA, AWWA, WEF, 24th Edition, 2023. Part 9221 B. |
| *E. coli* | 3.6 | 2.2 | 6.9 | 9.2 | 23 | 2.2 | <1.1 | - | MPN/ 100 mL | #APHA, AWWA, WEF, 24th Edition, 2023. Part 9221 F. |
