# Supplementary material for: Compounded environmental health risks in mountain communities upstream of Bhumibol Dam, Thailand
Source: PLoS One. 2026 Jun 9;21(6):e0340557. doi: 10.1371/journal.pone.0340557 (PMC13249165; doi:10.1371/journal.pone.0340557)
Supplement: Attachment — Submitted filename: Response to Reviewers - 5.5.2026.pdf [file pone.0340557.s004.pdf]

Response to Reviewers

1. Please revise: The abstract's subheadings could be included in a paragraph within 300 words.
Response page 2, lines 22-44: We have revised the abstract into a single, continuous paragraph of approximately 285 words. It now includes the required subheadings (Background, Methods, Results, Conclusion) within that paragraph as requested.

2. Please check the grammar throughout the paper; Table 1 is only an example.
Response pages 41, lines 738-743, and page 42 lines 752-758: We have conducted a thorough grammatical review of the entire manuscript. Specifically, regarding Table 1, we have reordered the villages to follow the consistent **V1–V6** sequence (Aum Haum to San Pa Puai) used in the text. We have also corrected the sampling dates in the Table 1 caption to **19–21 November 2024** to match the Methodology section.

3. Lines 154-156: Please add by purposive sampling. Additionally, samples from each water source were collected in two polyethylene bottles for pesticide detection and toxicity level assessment by purposive sampling.
Response page 7, lines 140-141: We have updated the Methodology (Lines 154–156) to include this phrase. The sentence now reads: "*Additionally, samples from each water source were collected in two polyethylene bottles for pesticide detection and toxicity level assessment* ***by purposive sampling****.*"

4. According to the abstract, this study assessed the water quality, usage patterns, and contamination pathways. It implies that the water quality is the outcome, and compounded environmental health risks are the factors to be detected.
Response We have refined the manuscript's terminology to ensure that water quality is framed as the primary outcome, while the compounded environmental health risks are treated as the factors being assessed, as follows:

- page 1, lines 24-29: The framing of "water quality" as the outcome and "usage patterns/contamination pathways" as the focus is located in the **Methods** section of the abstract. "*This study assessed the water quality, usage patterns, and contamination pathways across six villages upstream of Bhumibol Dam to shed light on the obstacles to sustainable water security. Samples from 38 water sources of drinking and/or non-drinking water, soil, and the edible parts of crops were subjected to analyses of physical, chemical (NO_3-N, pH), and qualitative pesticide-related variables, alongside a 6-month assessment of a community water filter system.*"
- page 5, lines 95-103: The explicit definition of "compounded environmental health risks" and the refined overarching goal (framing water quality as the outcome) is located at the end of the **Introduction**. "*Accordingly, this study was implemented with the aims of*

characterizing the quality of natural drinking water sources and water storage facilities used by the mountain communities upstream of Bhumibol Dam; investigating the pathways of agrochemical contamination into water, soil, and crops; and assessing the effectiveness of community water filtration systems. Additionally, compounded environmental health risks are defined here as the intersection of natural mineral-rich hydrogeology, agricultural chemical contamination, and local water-use behaviours. The overarching goal of this work is to enhance sustainable access to safe drinking water and improve health outcomes for these vulnerable communities."

- page 25, line 428-435: The reinforcement of this framing, specifically regarding the "compounding effect" and its outcome, is located in the **Discussion**. "*The second critical issue emerges from the intersection of monoculture farming and water safety, where the Principal Component Analysis (PCA) identifies a distinct at-risk cluster that exemplifies compounded environmental health risks. Rather than facing isolated issues, the communities in villages V2 and V3 are exposed to a triad of stressors: (1) high mineral toxicity indicated by elevated SPC and TDS; (2) high alkaline pH, and (3) significant nitrate (NO_3-N) and pesticide residuals. The outcome of this compounding effect is an elevated physiological burden on the residents, potentially manifesting in the high reported rates of urolithiasis observed in these specific clusters of the high-water quality.*"

5. The reviewer found that this statement has disappeared and was not found as you mentioned; please add this statement as suggested: "The outcome of this compounding effect is an elevated physiological burden on the residents, potentially manifesting in the high reported rates of urolithiasis observed in these specific clusters of the high water quality."
Response page 25, lines 433-435: We apologize for the omission in the previous version. We have re-inserted the exact sentence into the Discussion section under the subheading *"Evidence of pesticide contamination and associated risk"* following the description of the PCA stressors.

6. A new co-author is asked to be added for Krittiya Trivalairat... The request is unacceptable during the revision process and constitutes an ethical issue. Please remove.
Response We decline to remove Krittiya Trivalairat, as her contributions meet the authorship criteria defined by PLOS ONE.

7. Reviewer marked “No” to "Have the authors made all data underlying the findings in their manuscript fully available?

Response We have ensured that all underlying data required to support the study’s findings are included within the manuscript and its supporting information files. We remain committed to full transparency as per PLOS ONE’s data policy.
