# Supplementary material for: Compounded environmental health risks in mountain communities upstream of Bhumibol Dam, Thailand
Source: PLoS One. 2026 Jun 9;21(6):e0340557. doi: 10.1371/journal.pone.0340557 (PMC13249165; doi:10.1371/journal.pone.0340557)
Supplement: Attachment — Submitted filename: Response to Reviewers - 9.2.26 - ver.3.docx [file pone.0340557.s003.docx]

**Response to Reviewers**

0. If applicable, we recommend that you deposit your laboratory protocols in protocols.io to enhance the reproducibility of your results. Protocols.io assigns your protocol its own identifier (DOI) so that it can be cited independently in the future. For instructions see: https://journals.plos.org/plosone/s/submission-guidelines#loc-laboratory-protocols. Additionally, PLOS ONE offers an option for publishing peer-reviewed Lab Protocol articles, which describe protocols hosted on protocols.io. Read more information on sharing protocols at <https://plos.org/protocols?utm_medium=editorial-email&utm_source=authorletters&utm_campaign=protocols>.

**Response**: We deposited protocols in protocols.io as in this link below and cited in.

DOI: dx.doi.org/10.17504/protocols.io.4r3l2zpz3l1y/v1 (Private link for reviewers: https://www.protocols.io/private/1A03CEB912FF11F1B0580A58A9FEAC02 to be removed before publication.)

- On section “**Materials and Methods**” (Study location and water sources) (page 8, line 172-173), inserted “All protocols in this study were deposited in protocols.io (http://dx.doi.org/10.17504/protocols.io.4r3l2zpz3l1y/v1).”

1. Please ensure that your manuscript meets PLOS ONE's style requirements, including those for file naming. The PLOS ONE style templates can be found at

<https://journals.plos.org/plosone/s/file?id=wjVg/PLOSOne_formatting_sample_main_body.pdf>

and <https://journals.plos.org/plosone/s/file?id=ba62/PLOSOne_formatting_sample_title_authors_affiliations.pdf>

**Response**: Thank you for your recommendation. We have revised manuscript following the PLOS ONE style.

2. In your Methods section, please provide additional information regarding the permits you obtained for the work. Please ensure you have included the full name of the authority that approved the field site access and, if no permits were required, a brief statement explaining why.

**Response**:

- On section **“Materials and Methods”** (Study location and water sources) (page 6, line 123-128), inserted “Access to these field sites was granted by the Sam Ngao District Public Health Office (Tak Province, Thailand) under the “Promoting health and preventing water–borne disease of people living in the area of Bhumibol Dam” project. No additional collection permits were required as the study involved sampling from communal water sources and agricultural lands with the cooperation of local health authorities.”. As in “Attachment 1 - Area permission letter”.

3. We note that the grant information you provided in the ‘Funding Information’ and ‘Financial Disclosure’ sections do not match. When you resubmit, please ensure that you provide the correct grant numbers for the awards you received for your study in the ‘Funding Information’ section.

**Response**: We apologize for the discrepancy between the Funding Information and Financial Disclosure sections. We have now synchronized both sections to ensure all grant numbers and awarding bodies are consistent. We have updated the online submission metadata to match the manuscript text as follows:

- On section “**Financial Disclosure**”, informed “This research project received funding support from Chulabhorn Royal Academy (Grant number RF-CRA-2557-007).”
- On section “**Funding Information**”, informed “This research project received funding support from Chulabhorn Royal Academy (Grant number RF-CRA-2557-007). The funders had no role in study design, data collection and analysis, decision to publish, or preparation of the manuscript. There was no additional external funding received for this study.”

4. Thank you for stating in your Funding Statement:

“This research project received funding support from Chulabhorn Royal Academy.”

Please provide an amended statement that declares *all* the funding or sources of support (whether external or internal to your organization) received during this study, as detailed online in our guide for authors at http://journals.plos.org/plosone/s/submit-now. Please also include the statement “There was no additional external funding received for this study.” in your updated Funding Statement.

Please include your amended Funding Statement within your cover letter. We will change the online submission form on your behalf.

**Response**: We have updated the financial disclosure and the Role of Funder statement as requested.

- On section “**Funding Information**”, informed “This research project received funding support from Chulabhorn Royal Academy (Grant number RF-CRA-2557-007). The funders had no role in study design, data collection and analysis, decision to publish, or preparation of the manuscript. There was no additional external funding received for this study.”

5. Thank you for stating the following financial disclosure:

“This research project received funding support from Chulabhorn Royal Academy.”

Please state what role the funders took in the study. If the funders had no role, please state: "The funders had no role in study design, data collection and analysis, decision to publish, or preparation of the manuscript."

If this statement is not correct you must amend it as needed.

Please include this amended Role of Funder statement in your cover letter; we will change the online submission form on your behalf.

**Response**: We have updated the financial disclosure and the Role of Funder statement as requested.

- On section “**Funding Information**”, informed “This research project received funding support from Chulabhorn Royal Academy (Grant number RF-CRA-2557-007). The funders had no role in study design, data collection and analysis, decision to publish, or preparation of the manuscript. There was no additional external funding received for this study.”

6. Thank you for stating the following in the Acknowledgments Section of your manuscript:

“This research project received funding support from Chulabhorn Royal Academy. Access to YSI EXO multiparameter instruments was supported by the Department of Zoology, Faculty of Science, Kasetsart University. Additionally, we thank Tom Buckle from Scribendi ([www.scribendi.com](http://www.scribendi.com)) for editing a draft of this manuscript.”

We note that you have provided funding information that is currently declared in your Funding Statement. However, funding information should not appear in the Acknowledgments section or other areas of your manuscript. We will only publish funding information present in the Funding Statement section of the online submission form.

Please remove any funding-related text from the manuscript and let us know how you would like to update your Funding Statement. Currently, your Funding Statement reads as follows:

“This research project received funding support from Chulabhorn Royal Academy.”

Please include your amended statements within your cover letter; we will change the online submission form on your behalf.

This reviewer is asking you to clean up the manuscript by separating **financial support** (which goes into the journal's metadata) from **non-financial support** (which stays in the Acknowledgments).

PLOS ONE has a strict policy that funding details must not be in the body of the paper because they want to manage that information via the official "Funding Statement" to ensure it is searchable and standardized.

**Response**:

- We have revised on section “**Acknowledgement**” from “This research project received funding support from Chulabhorn Royal Academy. Access to YSI EXO multiparameter instruments was supported by the Department of Zoology, Faculty of Science, Kasetsart University. Additionally, we thank Tom Buckle from Scribendi ([www.scribendi.com](http://www.scribendi.com)) for editing a draft of this manuscript.”
  to “Access to YSI EXO multiparameter instruments was supported by the Department of Zoology, Faculty of Science, Kasetsart University. Additionally, we thank Tom Buckle from Scribendi ([www.scribendi.com](http://www.scribendi.com)) for editing a draft of this manuscript.”

7. We note that Figure 1 in your submission contain map/satellite images which may be copyrighted. All PLOS content is published under the Creative Commons Attribution License (CC BY 4.0), which means that the manuscript, images, and Supporting Information files will be freely available online, and any third party is permitted to access, download, copy, distribute, and use these materials in any way, even commercially, with proper attribution. For these reasons, we cannot publish previously copyrighted maps or satellite images created using proprietary data, such as Google software (Google Maps, Street View, and Earth). For more information, see our copyright guidelines: http://journals.plos.org/plosone/s/licenses-and-copyright.

We require you to either (1) present written permission from the copyright holder to publish these figures specifically under the CC BY 4.0 license, or (2) remove the figures from your submission:

a. You may seek permission from the original copyright holder of Figure 1 to publish the content specifically under the CC BY 4.0 license.

We recommend that you contact the original copyright holder with the Content Permission Form (http://journals.plos.org/plosone/s/file?id=7c09/content-permission-form.pdf) and the following text:

“I request permission for the open-access journal PLOS ONE to publish XXX under the Creative Commons Attribution License (CCAL) CC BY 4.0 (http://creativecommons.org/licenses/by/4.0/). Please be aware that this license allows unrestricted use and distribution, even commercially, by third parties. Please reply and provide explicit written permission to publish XXX under a CC BY license and complete the attached form.”

Please upload the completed Content Permission Form or other proof of granted permissions as an "Other" file with your submission.

In the figure caption of the copyrighted figure, please include the following text: “Reprinted from [ref] under a CC BY license, with permission from [name of publisher], original copyright [original copyright year].”

b. If you are unable to obtain permission from the original copyright holder to publish these figures under the CC BY 4.0 license or if the copyright holder’s requirements are incompatible with the CC BY 4.0 license, please either i) remove the figure or ii) supply a replacement figure that complies with the CC BY 4.0 license. Please check copyright information on all replacement figures and update the figure caption with source information. If applicable, please specify in the figure caption text when a figure is similar but not identical to the original image and is therefore for illustrative purposes only.

The following resources for replacing copyrighted map figures may be helpful:

USGS National Map Viewer (public domain): http://viewer.nationalmap.gov/viewer/

The Gateway to Astronaut Photography of Earth (public domain): http://eol.jsc.nasa.gov/sseop/clickmap/

Maps at the CIA (public domain): https://www.cia.gov/library/publications/the-world-factbook/index.html and https://www.cia.gov/library/publications/cia-maps-publications/index.html

NASA Earth Observatory (public domain): http://earthobservatory.nasa.gov/

Landsat: http://landsat.visibleearth.nasa.gov/

USGS EROS (Earth Resources Observatory and Science (EROS) Center) (public domain): http://eros.usgs.gov/#

Natural Earth (public domain): <http://www.naturalearthdata.com/>

**Response**: We have addressed copyright concerns regarding Figure 1. All geographic layers, including administrative boundaries and topographical features, were sourced from public domain databases, specifically [Department of Water Resources (DWR) GIS, GISTDA (Geo-Informatics and Space Technology Development Agency)]. No copyrighted satellite imagery or proprietary data from Google (Maps/Earth) were used.

- On section “**Materials and Methods**” (Study location and water sources) (page 6, line 130-131), inserted “Map data was retrieved from Department of Water Resources (DWR) GIS and Geo-Informatics and Space Technology Development Agency (GISTDA).”
- On “**Figure 1**” (page 8-9, line 176-179), revised from “**Fig. 1** Map of villages upstream of Bhumibol Dam, Sam Ngao District, Tak Province, Thailand.”
  to “**Fig. 1** Map of villages upstream of Bhumibol Dam, Sam Ngao District, Tak Province, Thailand. Map was created using QGIS (v 3.44) and retrieved data from Department of Water Resources (DWR) GIS and Geo-Informatics and Space Technology Development Agency (GISTDA).”

8. We notice that your supplementary table is included in the manuscript file. Please remove them and upload them with the file type 'Supporting Information'. Please ensure that each Supporting Information file has a legend listed in the manuscript after the references list.

**Response**: Already separated to other file “Supporting Information”.

- Page 28, line 550-553: Insert section “**Supporting Information**” **S1 Table.** Descriptive analysis of water quality parameters of samples from a community water filters (Village V3, Hin Lat), evaluated across three sampling points over 6 months at approximately 3-month intervals.

9. If the reviewer comments include a recommendation to cite specific previously published works, please review and evaluate these publications to determine whether they are relevant and should be cited. There is no requirement to cite these works unless the editor has indicated otherwise.

9.1 Is the manuscript technically sound, and do the data support the conclusions?

The manuscript must describe a technically sound piece of scientific research with data that supports the conclusions. Experiments must have been conducted rigorously, with appropriate controls, replication, and sample sizes. The conclusions must be drawn appropriately based on the data presented.

Reviewer #1: Yes

Reviewer #2: Partly

**Response**: On “**Discussion**” (Choice of drinking water and water quality heterogeneity) (page 24, paragraph 3, line 436-439), we have revised as follows:

- inserted “While this study did not directly measure the prevalence of urolithiasis among residents, the high levels of nitrate-nitrogen and alkaline pH observed in preferred drinking sources align with established environmental risk factors for renal calculi.”
- inserted “A limitation of this study is the brief sampling window. This period was selected to capture the water‑quality profile during the dry season, when agricultural activity (post‑harvest) and reliance on stagnant water sources are both high. Sampling at this time allows identification of compounded risks during a period of increased community vulnerability and when access to sand‑puddle sources is limited to the dry season.”

9.2 Has the statistical analysis been performed appropriately and rigorously?

Reviewer #1: Yes

Reviewer #2: No

**Response**: We have revised section “**Materials and Methods**” (Statistical analysis) (page 9-10, line 197-206) as follows:

- from “Normality tests conducted using the Shapiro–Wilk method indicated that most water quality parameters did not follow a normal distribution within each village (p < 0.05). Therefore, non-parametric statistical methods were employed, with Kruskal–Wallis tests conducted in SPSS 27.0 to compare differences among villages. To identify specific pairwise differences, Mann–Whitney U tests were performed as post hoc analyses. These tests were also applied to data collected from the water filter at Hin Lat at three different timepoints to assess the consistency and reliability of the water quality measurements over time.”
  to “Data were analyzed using SPSS 27.0. Before analysis, normality tests conducted using the Shapiro–Wilk method indicated that most water quality parameters did not follow a normal distribution within each village (p < 0.05). Therefore, non-parametric statistical methods were employed, with Kruskal–Wallis tests to compare differences among villages. To identify specific pairwise differences, Mann–Whitney U tests were performed as post hoc analyses. These tests were also applied to data collected from the water filter at Hin Lat at three different timepoints to assess the consistency and reliability of the water quality measurements over time.”

9.3 Have the authors made all data underlying the findings in their manuscript fully available?

The PLOS Data policy requires authors to make all data underlying the findings described in their manuscript fully available without restriction, with rare exception (please refer to the Data Availability Statement in the manuscript PDF file). The data should be provided as part of the manuscript or its supporting information, or deposited to a public repository. For example, in addition to summary statistics, the data points behind means, medians and variance measures should be available. If there are restrictions on publicly sharing data—e.g. participant privacy or use of data from a third party—those must be specified.

Reviewer #1: Yes

Reviewer #2: Yes

**Response**: We appreciate your decision. All data underlying the findings of this manuscript have been made fully available without restriction.

9.4 Is the manuscript presented in an intelligible fashion and written in standard English?

PLOS ONE does not copyedit accepted manuscripts, so the language in submitted articles must be clear, correct, and unambiguous. Any typographical or grammatical errors should be corrected at revision, so please note any specific errors here.

Reviewer #1: Yes

Reviewer #2: No

**Response**: In this manuscript, we used ChatGPT and Gemini in order to check the grammar and improve the language before editing by Tom Buckle from Scribendi ([www.scribendi.com](http://www.scribendi.com)).

9.5 Review Comments to the Author

Please use the space provided to explain your answers to the questions above. You may also include additional comments for the author, including concerns about dual publication, research ethics, or publi:cation ethics. (Please upload your review as an attachment if it exceeds 20,000 characters)

9.5.1 Reviewer #1: Congratulations to the authors, and nice work on the innovative study. I suggest importing the figures from SPSS 27V. In the manuscript are a few histograms and diagrams about the results of studies and demographic details.

**Response**: We have revised as follows:

- On section “**Materials and Methods**” (Statistical analysis) (page 9-10, line 199-201), insert “Box-and-whisker plots were used to visualize and analyze the spatial variation and statistical distribution of water quality variables across the six study villages.”
- Insert new “**Figure 4**”, and
- Revised former “**Fig. 4, 5, 6**” to new “**Fig. 5, 6, 7**”

9.5.2 Reviewer #2: ***Compounded environmental health risks need to be added for operational definitions and keywords.

**Response**: We have revised as follows:

- On section “**Keywords**” (page 3, line 55-57), revised former “pesticide” to “agrochemical runoff”, and inserted “hydrogeochemistry, water security, indigenous health”
- On section “**Introduction**” (page 5, paragraph 3, line 112-114), inserted “Additionally, compounded environmental health risks are defined here as the intersection of natural mineral-rich hydrogeology, agricultural chemical contamination, and local water-use behaviours”

9.5.3 Reviewer #2: ***The abstract’s conclusion is needed to add compounded environmental health risks and the solutions.

**Response**: We have revised on section “**Abstract**” (page 2-3, line 48-53) as follows:

- From “These findings reveal a severe, compounded public health threat of chronic exposure to minerals linked to urolithiasis and agrochemicals, highlighting the urgent need for quantitative risk assessment and the implementation of resilient, decentralized water treatment solutions in these mountain communities”
  to “Overall, these findings reveal compounded environmental health risks emerging from the convergence of mineral-rich hydrogeology, agrochemical runoff, and vulnerable water-collection behaviours. To mitigate these threats, the study suggests that decentralized water management strategies must prioritize both the implementation of advanced filtration technologies capable of mineral and chemical removal and targeted public health education to shift community reliance away from high-risk, stagnant water sources.”

9.5.4 Reviewer #2:***Please add compounded environmental health risks to the literature review, the outcome, and the conclusion.

**Response**: We have revised as follows:

- On section “**Introduction**” (page 5, paragraph 3, line 112-114), inserted “Additionally, compounded environmental health risks are defined here as the intersection of natural mineral-rich hydrogeology, agricultural chemical contamination, and local water-use behaviours”
- On section “**Discussion**” (Evidence of pesticide contamination and associated risk) (page 24-25, line 456-461), inserted “The PCA identifies a distinct 'at-risk' cluster that exemplifies compounded environmental health risks. Rather than facing isolated issues, the communities in villages V2 and V3 are exposed to a triad of stressors: (1) high mineral toxicity (indicated by elevated SPC and TDS), (2) high alkaline pH, and (3) significant nitrate and pesticide residuals. The outcome of this compounding effect is an elevated physiological burden on the residents, potentially manifesting in the high reported rates of urolithiasis observed in these specific clusters.”

9.5.5 Reviewer #2:***The abstract is required for the subheadings background, methods, results, and conclusion to ensure the required content is included.

**Response**: We appreciate the reviewer’s suggestion to include subheadings in the abstract for improved clarity. However, according to the PLOS ONE Submission Guidelines for the Abstract section, the journal requires the abstract to be a single, unstructured paragraph without subheadings, and within a 300-word limit. To address the reviewer’s underlying concern for clarity and structure, we have ensured that the abstract clearly follows a logical progression, from background and objectives to methods, key results, and the final conclusion, within the required single-paragraph format.

9.5.6 Reviewer #2:***A sampling technique is required for samples from 38 drinking and/or non-drinking water sources.

**Response**: We have revised on section “**Materials and Methods**” (Study location and water sources) as follows:

- Page 6, paragraph 1, line 140-142, revised from “Thirty-eight water sources were purposively selected as they represented the total land area accessible to and used by the community at the time of data collection.”
  to “All water sources in study areas were purposively selected as they represented the total land area accessible to and used by the community at the time of data collection.”, we designed to access on all water sources in this area, and there were only 38 sources on that time.
- Page 7, paragraph 2, line 154-156, inserted “Additionally, samples from each water source were collected in two polyethylene bottles for pesticide detection and toxicity level assessment.”

9.5.7 Reviewer #2:***A data collection period is required to cover a 6-month assessment. However, (on page 12), between 19 and 21 November 2024.

**Response**: We acknowledge the reviewer’s concern regarding the sampling duration. While the intensive field collection took place between 19 and 21 November 2024, this period was strategically selected as it represents the transition from the wet to the dry season in Northern Thailand. This window is critical for observing both residual agrochemical runoff from the late monsoon and the increasing mineral concentration/stagnation in sand-filtered puddles as water levels begin to recede. However, we recognize that a 6-month longitudinal study would provide deeper insights into seasonal variations. We have updated the Materials and Methods to clarify the rationale for this window and added a dedicated "Study Limitations" paragraph in the Discussion to address the need for future longitudinal monitoring.

- On section “**Materials and Methods**” (Study location and water sources) (page 6, paragraph 1, line 136-140), inserted “This period was selected to capture the water‑quality profile during the dry season, when agricultural activity (post‑harvest) and reliance on stagnant water sources are both high. Sampling at this time allows identification of compounded risks during a period of increased community vulnerability and when access to sand‑puddle sources is limited to the dry season.”
- On “**Discussion**” (Choice of drinking water and water quality heterogeneity) (page 24, paragraph 3, line 451-453), inserted “A limitation of this study is the brief sampling window. Future research involving longitudinal monitoring across both monsoon and dry seasons would provide more comprehensive view of seasonal agrochemical runoff patterns.”

9.5.8 Reviewer #2:***Please check grammar, such as Table 1, tested variables (not tested variable).

**Response**: We have revised on “**Supporting information**” (Supporting information file, 1^st^ column, 1^st^ row), revised from “Tested variable” to “Tested variables”

9.5.9 Reviewer #2:*** Please ensure that all related variables (test variables) are included in this study’s literature review, discussion, and conclusion.

**Response**: We have revised as follows:

- On section “**Introduction**” (page 3-4, paragraph 1, 60-82), from “Access to clean water is essential for human health, supporting hydration and overall body function, as well as enabling hygienic cooking and cleaning practices. According to the principles of the Ottawa Charter and the tenets upheld by the World Health Organization (WHO), access to clean drinking water is a fundamental component of public health and a basic human right that promotes health equity and well-being [1,2]. In rural areas, particularly those at high altitudes, communities depend on water sources derived from mountain for their daily life. This water is renowned for its purity and high mineral content, which can help to maintain balanced pH and support hydration, while also being essential for agriculture. However, the high levels of alkaline minerals such as calcium and magnesium in water in mountainous regions can also lead to health complications, including the formation of bladder stones [3–7]. Moreover, many rural communities around the world are facing challenges to their water security exacerbated by contamination (with herbicides, pesticides, and heavy metals) and climate change (such as long droughts and less precipitation), which has influenced the availability of clean fresh water. This necessitates the development of effective water management strategies to ensure that populations continue to have safe access to this vital resource [3,8–11]. Indeed, the responsible stewardship of mountain water is critical for safeguarding the well-being of mountain communities and securing their sustainable access to clean drinking water.”
  to “Access to clean water is essential for human health, supporting hydration and overall body function, as well as enabling hygienic cooking and cleaning practices. According to the principles of the Ottawa Charter and the tenets upheld by the World Health Organization (WHO), access to clean drinking water is a fundamental component of public health and a basic human right that promotes health equity and well-being [1,2]. In rural areas, particularly those at high altitudes, communities depend on water sources derived from mountain for their daily life. Water safety in these highland areas is governed by basic physicochemical parameters (temperature, pH, dissolved oxygen) and mineral content (specific conductance, total dissolved solids, salinity), which dictate both pathogen survival and the long-term risk of urolithiasis (bladder stones). This mountain water is renowned for its purity and high mineral content, which can help to maintain balanced pH and support hydration, while also being essential for agriculture. However, the high levels of alkaline minerals such as calcium and magnesium can also lead to health complications, including the formation of bladder stones [3–7]. Moreover, agricultural markers like nitrate-nitrogen (NO_3_-N) serve as critical indicators of anthropogenic runoff in these vulnerable watersheds. Beyond natural mineralization [8–10], many rural communities around the world are facing challenges to their water security exacerbated by contamination (with herbicides, pesticides, and heavy metals) and climate change (such as long droughts and less precipitation), which has influenced the availability of clean fresh water. This necessitates the development of effective water management strategies to ensure that populations continue to have safe access to this vital resource [3,11–14]. Indeed, the responsible stewardship of mountain water is critical for safeguarding the well-being of mountain communities and securing their sustainable access to clean drinking water.”
- On section “**References**”, revised by
  - moved “53, 54, 56” to “8, 9, 10”
  - moved “8–52” to “11–55”
  - moved “55” to “56”

9.5.10 Reviewer #2:*** The discussion is required to reflect the study’s results in thematic analysis.

**Response**: We have revised on section “**Discussion**” as follows:

- On subsection “**Choice of drinking water and water quality heterogeneity**”
  - Page 24, line 436-439, inserted “While this study did not directly measure the prevalence of urolithiasis among residents, the high levels of nitrate-nitrogen and alkaline pH observed in preferred drinking sources align with established environmental risk factors for renal calculi.”
  - Page 24, line 447-450: inserted “The evidence suggests that geological profile of the region is not an isolated variable but a constant environmental stressor that compounds the risk of urolithiasis through the community’s specific choice of highly mineralized drinking sources.”
    - On subsection “**Evidence of pesticide contamination and associated risk**”
  - Page 24-25, line 456-461, inserted “The second critical issue emerges from the intersection of monoculture farming and water safety, where the Principal Component Analysis (PCA) identifies a distinct at-risk cluster that exemplifies compounded environmental health risks. Rather than facing isolated issues, the communities in villages V2 and V3 are exposed to a triad of stressors: (1) high mineral toxicity indicated by elevated SPC and TDS; (2) high alkaline pH, and (3) significant nitrate (NO_3_-N) and pesticide residuals.”
  - Page 25, line 466-469, revised “The strong correlation observed in the PCA between the NO3N and TOX vectors is a convincing indication of a common, diffuse source of contamination, which is clearly consistent with agricultural run-off (e.g., of agricultural chemicals) from non-point sources.”
    to “The strong correlation between NO3N and TOX in preferred sources, such as sand–filtered puddles, identifies agricultural runoff (e.g., of agricultural chemicals) as a dominant anthropogenic stressor.”
- On subsection “**Inadequate water infrastructure and public health implications**”
  - Page 26-27, line 506-510, inserted “Behavioral and technical dimensions of water security address a vulnerability gap where residents often prefer untreated, stagnant sources, such as sand–filtered puddles, over community–provided systems due to accessibility or perceived taste. This suggests that health risks are not only environmental but are exacerbated by a mismatch between community needs and decentralized water treatment capabilities.”
  - Page 27, line 516-518, revised “This directly supports the conclusion that the filter does not function effectively at removing contaminants and ensuring a supply of clean, potable water.”
    to “This directly supports the conclusion that the filter does not function effectively at removing compounded risks of high mineralization and chemical runoff, leaving the community under the misapprehension that their water supply is safe.”

9.6 PLOS authors have the option to publish the peer review history of their article (what does this mean?). If published, this will include your full peer review and any attached files.

If you choose “no”, your identity will remain anonymous but your review may still be made public.

Do you want your identity to be public for this peer review? For information about this choice, including consent withdrawal, please see our Privacy Policy.

Reviewer #1: No

Reviewer #2: No

10. Other responses

**Response**: We have added a new co-author (Krittiya Trivalairat) from her helping in laboratory, drafting manuscript, and revising process.

[NOTE: If reviewer comments were submitted as an attachment file, they will be attached to this email and accessible via the submission site. Please log into your account, locate the manuscript record, and check for the action link "View Attachments". If this link does not appear, there are no attachment files.]

To ensure your figures meet our technical requirements, please review our figure guidelines: https://journals.plos.org/plosone/s/figures

You may also use PLOS’s free figure tool, NAAS, to help you prepare publication quality figures: https://journals.plos.org/plosone/s/figures#loc-tools-for-figure-preparation.

NAAS will assess whether your figures meet our technical requirements by comparing each figure against our figure specifications.
